# Evaluation for type 1 diabetes associated autoantibodies in diabetic and non-diabetic Australian terriers and Samoyeds

**DOI:** 10.1186/s40575-020-00089-5

**Published:** 2020-08-11

**Authors:** Allison L. O’Kell, Clive H. Wasserfall, Paula S. Henthorn, Mark A. Atkinson, Rebecka S. Hess

**Affiliations:** 1 Department of Small Animal Clinical Sciences, College of Veterinary Medicine, The University of Florida, 2015 SW Archer Rd, Gainesville, FL 32608 USA; 2 Department of Pathology, Immunology, and Laboratory Medicine, College of Medicine, The University of Florida Diabetes Institute, 1275 Center Dr., Gainesville, FL 32610 USA; 3 Department of Clinical Studies, School of Veterinary Medicine, University of Pennsylvania, 3900 Delancey St., Philadelphia, PA 19104 USA; 4 Department of Pediatrics, College of Medicine, The University of Florida Diabetes Institute, 1275 Center Dr., Gainesville, FL 32610 USA

**Keywords:** Canine, Diabetes, Autoantibodies

## Abstract

**Background:**

Evidence for an autoimmune etiology in canine diabetes is inconsistent and could vary based on breed. Previous studies demonstrated that small percentages of diabetic dogs possess autoantibodies to antigens known to be important in human type 1 diabetes, but most efforts involved analysis of a wide variety of breeds. The objective of this study was to evaluate the presence of glutamic acid decarboxylase 65 (GAD65), insulinoma-associated protein 2 (IA-2), and zinc transporter 8 (ZnT8) autoantibodies in diabetic and non-diabetic Australian Terriers and Samoyeds, two breeds with comparatively high prevalence of diabetes, in the United States.

**Results:**

There was no significant difference in the proportion of samples considered positive for GAD65 or ZnT8 autoantibodies in either breed evaluated, or for IA-2 autoantibodies in Australian Terriers (*p* > 0.05). The proportion of IA-2 autoantibody positive samples was significantly higher in diabetic versus non-diabetic Samoyeds (*p* = 0.003), but substantial overlap was present between diabetic and non-diabetic groups.

**Conclusions:**

The present study does not support GAD65, IA-2, or ZnT8 autoantibodies as markers of autoimmunity in canine diabetes in Samoyeds or Australian Terriers as measured using human antigen sandwich enzyme-linked immunosorbent (ELISA) assays. Future studies using canine specific assays as well as investigation for alternative markers of autoimmunity in these and other canine breeds are warranted.

## Plain English summary

Diabetes mellitus represents one of the most common endocrine diseases in dogs, with certain breeds having a markedly higher risk for developing the disorder. Most dogs with diabetes have a deficiency in the production of insulin, a hormone produced by the pancreas that reduces blood sugar, due to a loss of the insulin producing cells in the pancreas (β-cells). The reason for this loss of β-cells is unknown, but previous studies have suggested that the body’s immune system may attack the pancreas in a process called autoimmune destruction. It is well known that autoimmune destruction of the β-cells leads to insulin deficiency in humans with Type 1 diabetes and special blood markers of this autoimmune response, termed autoantibodies, can be detected. These autoantibodies have only been infrequently detected in dogs with diabetes at this time. Yet, most studies have not evaluated the presence of these autoantibodies from large numbers of dogs of breeds with high risk for diabetes, and it is possible that some breeds may have an autoimmune cause for diabetes while others do not. In this study, we measured three different autoantibodies common to human type 1 diabetes (glutamic acid decarboxylase 65 (GAD65), insulinoma-associated protein 2 (IA-2), and zinc transporter 8 (ZnT8)) in a large group of diabetic and non-diabetic Samoyeds and Australian Terriers, two breeds at high risk for diabetes in the United States. An assay commonly performed in human diabetic patients was used in this study. We did not observe that diabetic dogs of either breed were more likely to be positive for any of the autoantibodies than non-diabetic dogs, but the use of the human assay does have some limitations. Our results do not provide support for these specific autoantibodies as markers of autoimmune diabetes using the humans assays in these two breeds. However, studying alternative blood markers of autoimmunity, or developing canine specific assays, may lead to additional information as to the role such mechanisms may have in the development of diabetes in dog.

## Background

Diabetes mellitus is a common endocrine disease in dogs, with the most frequent presentation being that of insulin deficiency with a life-long requirement for exogenous insulin [1]. Contributing factors to canine diabetes pathogenesis may include autoimmunity, pancreatitis, insulin resistance related to endocrine disease or high progesterone, and rarely, congenital β-cell hypoplasia [2].

Canine diabetes is considered to be similar to human type 1 diabetes (T1D) [1]; however, some important unknowns persist with respect to the disease pathogenesis in dogs that are well established in the human form of the disease. Human T1D is considered a chronic autoimmune disease characterized by islet autoimmunity and insulin deficiency [3]. Autoantibodies readily detectable in serum provide a marker of autoimmunity months to years before disease onset [3], with some 90–95% of patients positive for one or more autoantibodies at diagnosis [4]. Hence, autoantibodies are well established biomarkers in T1D, especially those that target insulin, glutamic acid decarboxylase 65 (GAD65), insulinoma associated protein 2 (IA-2), and zinc transporter 8 (ZnT8) [3, 5]. In contrast, evidence for autoimmune mechanisms, including the presence of autoantibodies as biomarkers in canine diabetes are inconsistent [2, 6]. Studies to date have noted insulin autoantibodies in 3–12.5% of untreated diabetic dogs [7, 8]. GAD65 autoantibodies were detected in 0.8–13% of diabetic dogs using a canine specific assay [9, 10], and in 0% of diabetic dogs and 20% of control dogs using a human assay [11]. Autoantibodies to IA-2 were observed in 10% of diabetic dogs using a canine specific assay [10] and 0% of diabetic dogs using a human assay [11]; the latter study also found no diabetic or control dogs to be positive for ZnT8 autoantibodies using a human assay.

The role of genetics in human T1D is well established, with human leukocyte antigen (HLA) haplotypes as the major genetic risk factor, along with over 60 non-HLA genes having lesser contributions [12]. With respect to canine diabetes, a variety of breeds have been found to have a higher risk of diabetes in various geographic regions [13–16], suggesting a genetic component to the disease in this species as well. The Samoyed has repeatedly been identified as a high risk breed in the United States, United Kingdom, and Sweden [13–16]. Odds ratios for developing the disease in Samoyeds in the United States were reported as 3.3 [13] and 11.8 [16], and in the United Kingdom as 35 [14]. In addition, in one study in the United States, Australian Terriers were noted as being 32 times more likely to develop diabetes compared with mixed breed dogs [13]. Additionally, Australian Terriers were also found to be at higher risk in Sweden [15] and Australia [17]. A variety of studies have identified genetic risk factors associated with the disease in multiple dog breeds, including dog leukocyte antigen (DLA) haplotype [18], T-cell cytokine gene polymorphisms [19], and CTLA4 gene polymorphisms [20]. These genetic associations often appear to be breed specific, and the degree of contribution of the identified genes to disease risk is currently unclear and may differ among breeds [14].

Hence, it has become increasingly apparent that canine diabetes is a heterogenous disease with multiple pathogenic factors [2, 6]. We have previously speculated that autoimmune mechanisms may be a component of the disease in a subset of diabetic dogs, potentially in certain breeds [6]. Studies investigating immune system involvement in canine diabetes thus far have generally included a wide variety of breeds, whereas studies interrogating potential autoimmune mechanisms in a large number of dogs from a single breed are lacking. As noted earlier, the Australian Terrier and Samoyed are of particular interest due to their previously documented high risk for developing diabetes [13–17]. Therefore, the objective of this study was to evaluate the presence of GAD65, IA-2, and ZnT8 autoantibodies in diabetic and non-diabetic Australian Terriers and Samoyeds geographically located in the United States using human antigen sandwich enzyme-linked immunosorbent (ELISA) assays.

## Results

### Study population

One hundred and thirty four (63 diabetic, 71 non-diabetic) Australian Terrier and 73 Samoyed (31 diabetic, 42 non-diabetic) serum samples were available for analysis. Age at sample collection was available for 61/63 diabetic and 58/71 non-diabetic Australian Terriers, and for 30/31 diabetic and 32/42 non-diabetic Samoyeds. The diabetic Australian Terriers were significantly older (mean ± SD = 10.7 ± 3 years) than the non-diabetic Australian Terriers (9.45 ± 2.2 years) (*p* = 0.003). There was no significant difference in age between diabetic (11.4 ± 2.6 years) and non-diabetic (10.6 ± 2.2 years) Samoyeds (*p* = 0.18). There was no significant difference in sex distribution between diabetic and control groups within Australian Terrier (*p* = 0.10) or Samoyed (*p* = 0.22) breed groups (Table 1). The median duration of diabetes in Australian Terriers for which data was available (58/63) was 2.5 years (range, 0–7.5 years) and in Samoyeds for which data was available (30/31) was 3 years (range, 0.9–9 years) (*p* = 0.68). Only eight Australian Terriers and six Samoyeds had a duration of disease less than or equal to one year.

**Table 1:** Sex Distribution in Australian Terrier and Samoyed Groups

Sex	Australian Terrier Diabetic n (%)	Australian Terrier Non-Diabetic n (%)	Samoyed Diabetic n (%)	Samoyed Non-Diabetic n (%)
Female Spayed	28 (44)	30 (42)	13 (42)	14 (33)
Female Intact	1 (2)	5 (7)	3 (10)	4 (10)
Male Neutered	30 (48)	26 (37)	12 (39)	16 (38)
Male Intact	2 (3)	8 (11)	0 (0)	5 (12)
Unknown	2 (3)	2 (3)	3 (10)	3 (7)
Total	63	71	31	42

### Total immunoglobulin G (IgG)

IgG was detected in all samples, with a median of 2467 mg/dL (range 336–10,453 mg/dL). Median total IgG in the diabetic group for both breeds combined (2500 mg/dL; range 336–10,204) was not different than the non-diabetic group for both breeds combined (2467 mg/dL; range 914–10,453) (*p* = 0.34).

### GAD65, IA-2, and ZnT8 autoantibody ELISAs

**Fig. 1:**
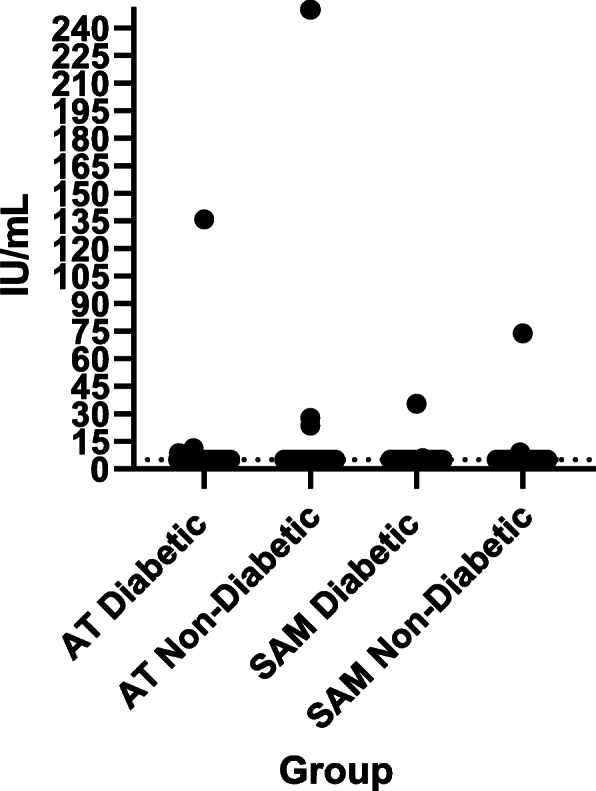
GAD65 Autoantibody ELISA. Results for GAD65 autoantibody ELISA in IU/mL. The dotted line represents the lower limit of quantification (5 IU/mL). AT = Australian Terrier; SAM = Samoyed

**Fig. 2:**
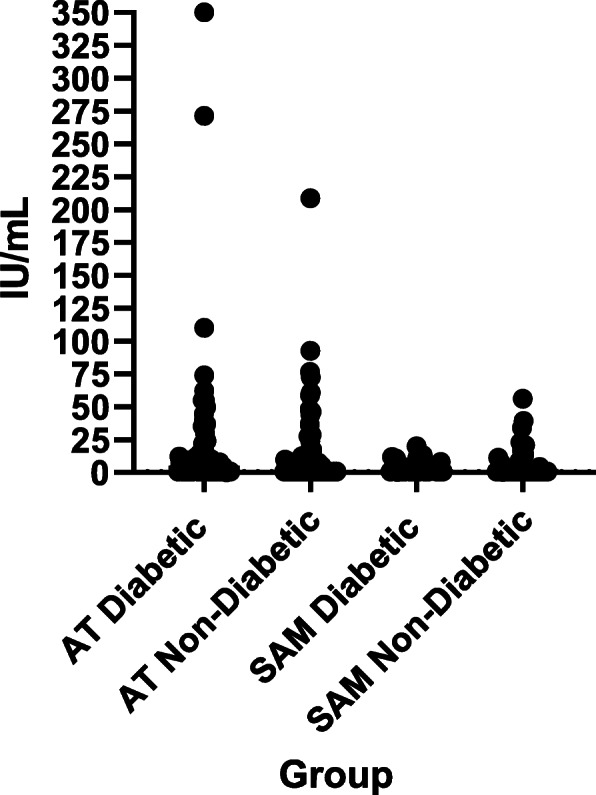
IA-2 Autoantibody ELISA. Results for IA-2 autoantibody ELISA in IU/mL. The lower limit of quantification is 0.75 IU/mL. AT = Australian Terrier; SAM = Samoyed

**Fig. 3:**
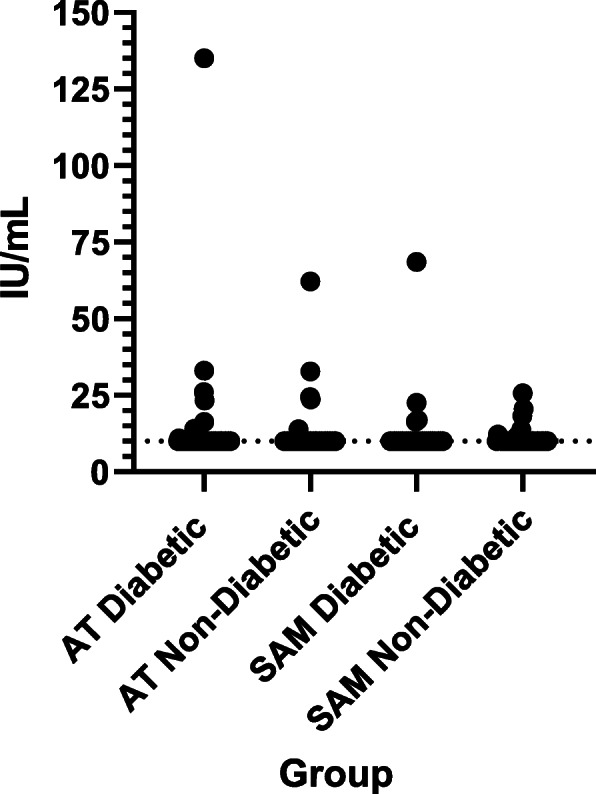
ZnT8 Autoantibody ELISA. Results for ZnT8 autoantibody ELISA in IU/mL. The dotted line represents the lower limit of quantification (10 IU/mL). AT = Australian Terrier; SAM = Samoyed

For GAD65 autoantibodies, the majority of samples in both Australian Terrier and Samoyed diabetic and non-diabetic groups were below level of quantification (and therefore considered to be “negative”). For Australian Terriers, 4/63 (6%) of diabetic and 4/71 (6%) of non-diabetic samples were above the level of quantification (and therefore considered to be “positive”) (*p* > 0.99) (Fig. 1). In Samoyeds, 2/31 (6%) of diabetic and 2/42 (5%) of non-diabetic samples were above the level of quantification (p > 0.99) (Fig. 1). There was marked overlap in IA-2 autoantibody results between diabetic and non-diabetic groups in both breeds. In Australian Terriers, 36/71 (51%) of diabetic and 33/71 (46%) of non-diabetic samples were above the level of quantification (*p* = 0.2), while in Samoyeds 19/31 (61%) of diabetic and 12/42 (29%) of non-diabetic samples were above the level of quantification (*p* = 0.003) (Fig. 2). For ZnT8, 8/63 (13%) of diabetic and 6/71 (8%) of non-diabetic Australian Terrier samples were above the limit of quantification (*p* = 0.342) (Fig. 3). In Samoyeds, 4/31 (13%) of diabetic and 7/42 (17%) of non-diabetic samples were above the ZnT8 limit of quantification (*p* = 0.75) (Fig. 3).

## Discussion

This study investigated the presence of autoantibodies against GAD65, IA-2, and ZnT8 in a large number of diabetic and non-diabetic Australian Terriers and Samoyeds, which are two of the breeds with the highest risk for diabetes in the United States [13, 16]. With the exception of IA-2 in Samoyeds, autoantibodies were not more frequently detectable in diabetic dogs versus non-diabetic control dogs of either breed. Although IA-2 was detected more frequently in Samoyeds with diabetes, many non-diabetic dogs also had detectable IA-2 autoantibodies, and the clinical relevance of this result is questionable. A limitation of this study is that the cutoff value for “positive” results in dogs has not been determined. We elected to consider any value above the limit of quantification, based on the lowest ELISA standard, as positive to maximize our ability to detect possible positive results. A limitation of this is that the relatively high frequency of detectable low level autoantibody titers for IA-2 in both diabetic and non-diabetic groups may be a result of non-specific binding. Both objective and subjective assessment of the data suggest that diabetic dogs do not have a clinically relevant increase in the frequency of detectable autoantibodies to GAD65, IA-2, or ZnT8 compared with non-diabetic controls dogs using these assays.

As an antigen sandwich assay, the ELISA methodology used in the present study should capture canine antibodies provided the canine antibody recognizes the epitope. Even so, one possible explanation for general lack of difference between diabetic and non-diabetic dog results is that the ELISA used was developed for and has only been validated in humans. However, Davison et al [10] sequenced canine GAD65 and IA-2 and found that the amino acid sequences were very similar between humans and dogs. Ahlgren et al [9] used immunoprecipitation to evaluate for GAD65 autoantibodies in a variety of dog breeds using both human and canine proteins and found similar results for both assays, with one positive diabetic dog using the canine protein and one positive control dog using the human protein. In both studies, known GAD65 autoantibody positive human serum remained positive using canine specific assays [9, 10]. Of note, Davison et al [10] found that reactivity for the control human positive serum for GAD65 using the canine assay was much higher than the canine positive cases, and the control human negative for both GAD65 and IA2 was much lower than most of the negative canine cases. Additionally, Davison et al. [10] also state that unpublished data from a pilot study identified high reactivity in control dogs using human radio-precipitation assays for GAD65 and IA2. These findings indicate that both false positive results (due to non-specific binding) or false negative results (if detection thresholds for canine autoantibody positive are lower than the ELISAs can quantify) are possible. Development of an optimized canine specific assay for the three autoantibodies studied here would be required to fully investigate these possibilities, and our results should be interpreted in light of these known limitations.

The natural history of autoantibody development is well described in human T1D, with insulin or GAD65 autoantibodies typically appearing first in childhood and IA-2 and ZnT8 often occurring closer to disease onset and in combination with other autoantibodies [21]. Positivity for GAD65 and IA-2 autoantibodies decreases over time in humans following T1D onset [22]. It is possible that more diabetic dogs in the study would have tested positive for autoantibodies earlier in life, especially given that all but 8 Australian Terriers and 6 Samoyeds had a duration of diabetes greater than 1 year at the time of sample collection. An additional possibility is that the non-diabetic dog group included dogs that were in a pre-diabetic or asymptomatic state and would later develop overt diabetes. This represents an additional limitation of the study as follow up data was not available for dogs in this study to verify the absence of diabetes throughout the remainder of their lives.

It is possible that an immunologic basis for diabetes is simply not common in dogs. Previous studies have yielded inconsistent results with respect to humoral and cellular autoimmunity as well as histologic assessment of insulitis, and this topic has been thoroughly reviewed recently [2, 6]. On the contrary, the relevant antigens in dogs may simply be different from those of human T1D, and accordingly, the autoantibodies important in the canine disease may not yet have been identified. Antibodies to additional novel autoantigens along with immune responses to post-translationally modified antigens and hybrid peptide antigens have been detected in humans with T1D [23]. The goal of these discoveries is not necessarily to replace the well-established autoantibody markers, but to provide information on pathogenesis of the disease as well as potential therapeutic targets [23]. Novel and unbiased approaches to autoantibody detection in dogs may yield findings specific to the disease in this species and should be pursued in future studies.

## Conclusions

In conclusion, the present study did not provide support that autoantibodies to GAD65, IA-2, or ZnT8 are markers of autoimmunity in diabetic Samoyeds and Australian Terriers using a common ELISA for these autoantibodies in human T1D. The potential for both false positive and negative results using these human assays is an important limitation. However, given the lack of supportive data in other studies regarding a potential role for these autoantibodies as biomarkers of canine diabetes, future studies should focus on searching for novel autoantigens and evidence of cellular autoimmunity as evidence of an immune mediated pathogenesis for canine diabetes, in addition to the development of canine specific assays to detect autoantibodies.

## Methods

### Study population

Dogs included in the present study were originally recruited as part of a study evaluating the insulin gene and diabetes in Australian Terriers and Samoyeds located in the United States [24] between January 2006 and December 2009. The University of Pennsylvania Privately Owned Animal Protocol Committee approved the study protocol and client consent form, and all owners provided consent prior to study participation. Diabetic dogs (cases) were classified based on owner and primary veterinarian confirmation of diabetes mellitus and therapy with insulin. Non-diabetic dogs (controls) were classified based on owner and primary veterinarian confirmation of lack of clinical signs or diagnosis of diabetes mellitus. Dog owners completed a questionnaire providing information about the dog’s age, sex (and if neutered), health status, and date of diabetes diagnosis (if applicable).

Blood samples were predominantly collected by the primary care veterinarian and shipped overnight to the University of Pennsylvania School of Veterinary Medicine in serum separator tubes or red top tubes. Some blood samples were collected at Samoyed breed club events or from the patient population at the University of Pennsylvania School of Veterinary Medicine. Serum was separated on arrival to the University of Pennsylvania and stored at − 80 °C until analysis.

### ELISAs

Due to the age of the stored samples, total IgG was quantified using a canine specific ELISA kit (Immunology Consultants Laboratory, Inc., Portland, OR) according to the manufacturer’s instructions to confirm that IgG was present. All samples were measured in duplicate. Autoantibodies for GAD65, IA-2, and ZnT8 were measured using commercial antibody kits from Kronus Inc. (Boise, ID, USA) that have been validated for use in human T1D autoantibody screening [25]. This assay is designed as an antigen sandwich assay and is therefore, isotype and species independent, provided that the canine antibody recognizes the epitope. Autoantibodies were measured in IU/mL. Results that were below or above the lower and upper limit of quantification based on the lowest and highest assay standard were set at the lower and upper limit for data analysis purposes: GAD (5 IU/mL, 250 IU/mL), IA-2 (0.75 IU/mL, 350 IU/mL), ZnT8 (10 IU/mL, 500 IU/mL).

### Statistical analysis

Data were tested for normality using the D’Agostino & Pearson test, and parametric or non-parametric tests were used as indicated. Age was compared for diabetic and non-diabetic groups within each breed using an unpaired t-test with Welch’s correction. Duration of diabetes was compared between breed groups with a Mann-Whitney U test. Sex distribution within each breed group was compared using a Chi squared test. IgG concentrations were compared between diabetic and control groups using a Mann Whitney U test. Given that the autoantibody concentration cutoffs for definitive positivity are unknown for dogs, the proportion of autoantibody ELISA results above the lower limit of quantification (“positive” samples) were compared between diabetic and non-diabetic groups for each autoantibody within each breed using a Chi squared or Fisher’s exact test. Statistical analysis was performed using GraphPad Prism software v8.0 (GraphPad, San Diego, CA).

## Data Availability

The datasets analysed during the current study are available from the corresponding author on reasonable request.

## Abbreviations

GAD65: glutamic acid decarboxylase 65
IA-2: insulinoma-associated protein 2
ZnT8: zinc transporter 8
T1D: type 1 diabetes
HLA: human leukocyte antigen
DLA: dog leukocyte antigen
CTLA4: cytotoxic T-lymphocyte-associated protein 4
IgG: immunoglobulin G
